# Pancytopenia in a patient with cystinosis secondary to myelosuppression from cystine crystal deposition: a case report

**DOI:** 10.1186/s13256-015-0691-8

**Published:** 2015-09-17

**Authors:** Yung Lyou, Xiaohui Zhao, Chaitali S. Nangia

**Affiliations:** 1 Department of Medicine, Division of Hematology Oncology, University of California Irvine Medical Center, 101 The City Drive South, Orange, CA 92868 USA; 2 Department of Pathology, University of California Irvine Medical Center, 101 The City Drive South, Orange, CA 92868 USA

**Keywords:** Cystinosis, pancytopenia

## Abstract

**Introduction:**

Cystinosis is a rare metabolic genetic disorder caused by a mutation in the cystinosin lysosomal cystine transporter gene. Clinically, it is characterized by systemic accumulation of cystine crystals in tissues causing end-organ dysfunction in the kidney, eyes, muscles, and other organs in the body. In very rare cases, it can also involve the bone marrow and the resulting cystine crystal deposition can cause myelosuppression leading to pancytopenia.

**Case presentation:**

Here we report the case of a 26-year-old white woman with cystinosis and other complex medical comorbidities who developed pancytopenia. She was worked up extensively and ruled out for common causes of pancytopenia (infectious disorders, vitamin deficiencies secondary to gastrointestinal malabsorption, rheumatologic, and hematologic disorders). On bone marrow biopsy she was found to have extensive deposits of cystine crystals, which was thought to be the cause of her myelosuppression leading to her pancytopenia. As a result, by treating her underlying cystinosis more aggressively we were able to observe an improvement in her pancytopenia a few months afterwards.

**Conclusions:**

Pancytopenia secondary to myelosuppression from cystine crystal deposition in the bone marrow is a very rare complication that has been reported in only a handful of case reports. This case illustrates the importance of keeping a broad differential diagnosis and systematically ruling out common causes of pancytopenia. It also demonstrates the importance of bone marrow biopsies in the evaluation of unexplained pancytopenia.

## Introduction

Cystinosis is a rare metabolic genetic disorder caused by a mutation in the cystinosin lysosomal cystine transporter (*CTNS*) gene characterized by an accumulation of cystine crystals in the tissues causing end-organ damage [1, 2]. This gene has been mapped to chromosome 17p13 [2]. It consists of 12 exons and encodes for 367 amino acids with a 65kb deletion being the most common gene mutation amongst these patients [2]. A study done in France, estimated that it affected 1 in 100,000 to 200,000 children worldwide at birth [3]. These patients most often develop end-organ dysfunction of the kidneys, eyes, and endocrine system with a mean life expectancy of 28.5 years [1, 3]. In rare cases patients will also develop complications of pancytopenia secondary to bone marrow suppression from cystine crystal deposition [4–9]. Here we report a rare case of pancytopenia in a patient with cystinosis secondary to myelosuppression from cystine crystal deposition.

## Case presentation

A 26-year-old white woman with hereditary cystinosis and multiple medical comorbidities was referred to us for newly developed pancytopenia. At 13 months she presented with nausea, vomiting, failure to thrive, and renal failure and required in-patient hospitalization. During this initial work up she was found to have hereditary cystinosis by detecting elevated levels of cystine in her peripheral blood leukocytes. It was determined that her cystinosis caused her to develop a secondary renal Fanconi’s syndrome, which had led to the symptoms of nausea, vomiting, failure to thrive, and renal failure. As a result, she was then started on cysteamine treatment as a young child. She then progressively developed worsening nephropathy secondary to her cystinosis and underwent a renal transplant at age 16. Three years later she unfortunately developed rejection of her transplanted kidney and required a nephrectomy. She was then put on chronic hemodialysis for end-stage renal disease (ESRD) three times a week. Due to her ESRD she developed chronic anemia secondary to chronic kidney disease (CKD). She eventually required several blood transfusions and had to be placed on weekly injections of erythropoietin. Over the next 4 years she then developed other medical conditions such as hypertension, hypothyroidism, esophagitis with esophageal ulceration status post-Nissen fundoplication, gastric outlet obstruction status post-Roux-en-Y surgery, and multiple deep vein thromboses requiring chronic anticoagulation with Coumadin (warfarin). Later she developed thrombocytopenia with platelets fluctuating between 40×10^3^/μl and 80×10^3^/μl. She was then referred to our clinic for a consult after a recent hospitalization found her to be pancytopenic with leukopenia, anemia, and thrombocytopenia.

At the time of the consult, she had stable vital signs and no clinical signs of bleeding. She denied any hematuria, melena, blood in her stools, or vaginal bleeding. Her physical examination was unremarkable with no signs of ecchymosis or contusions. Her complete blood count (CBC) showed white blood cell (WBC) count was 1.7×10^3^/μl with an absolute neutrophil count of 714/μl, a hemoglobin level of 10.0g/dl, hematocrit of 29.8%, and a platelet count of 38×10^3^/μl. Her mean corpuscular volume (MCV) was 84.8fl (normal range, 81.5 to 97.0fl). Her reticulocyte count was 2.4% (normal range, 0.9 to 2.5%) with a reticulocyte index of 1.18. Her vitamin B12 was 1298pg/ml (normal range, 180 to 1241pg/ml) and folate 8.9ng/ml (3.0 to 18.2ng/ml). She was taking Coumadin (warfarin) due to a history of recurrent deep vein thrombosis (DVT) so her prothrombin time and international normalized ratio (INR) were elevated at 28.2 seconds (normal range, 10.0 to 11.3 seconds) and 2.74 (target range, 2.0 to 3.0 for DVT treatment) respectively. She had been receiving erythropoietin for her anemia secondary to CKD so it was not measured. Her hepatitis B and C, parvovirus B16, and human immunodeficiency virus (HIV) assays were all negative. Her Epstein–Barr virus (EBV) antibody titers were positive but she did not have clinical signs of active mononucleosis such as fatigue, myalgia, fever, or sore throat. We then performed a peripheral blood smear which found her red blood cells (RBCs) to be hypochromatic and normocytic. Polychromasia was not increased while her WBCs and platelets were moderately decreased in numbers but normal in appearance. No circulating blasts or abnormal cells were visualized.

**Fig. 1:**
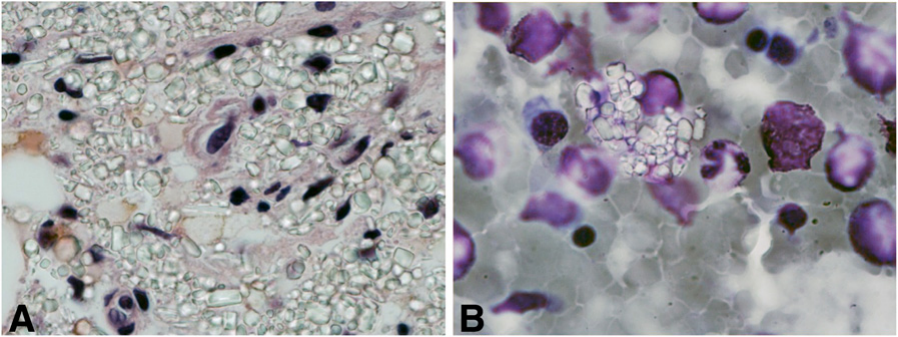
Bone marrow core and aspirate smear. Numerous rhomboid and rectangular shaped, birefringent cystine crystals are visualized under polarized light. **a** Sheets of histiocytes with refractile cystine crystals seen in the core biopsy (hematoxylin and eosin stain, ×400). **b** A single histiocyte contains multiple refractile rhomboid/rectangular crystals in the cytoplasm (Giemsa stain, ×1000)

We then performed a bone marrow biopsy. The bone marrow biopsy showed that the cellularity was 5% with active trilineage hematopoiesis with only a moderate amount of fat and stroma present. No circulating blasts or abnormal cells were visualized in the bone marrow biopsy aspirate or core. No immature cells were visualized and the myeloid to erythroid ratio was estimated to be 3 to 4:1. The megakaryocytes that were visualized appeared to be normal in appearance. The quantity of observed megakaryocytes were found to be decreased but adequate in number when one accounted for the overall panhypoplasia present in the examined biopsy specimen. Flow cytometry revealed no abnormalities and iron staining revealed increased iron stores. However, much to our surprise there were extensive sheets of deposits of rhomboid and rectangular shaped, birefringent cystine crystals visualized under polarized light, which occupied about 30% of the marrow components (Fig. 1). Since other causes of pancytopenia had been ruled out (infectious, gastrointestinal malabsorption, rheumatologic, and hematologic disorders), we were left with the most likely possibility being that she had myelosuppression/marrow replacement from deposition of cystine crystals as the cause of her pancytopenia. On further interview the patient revealed that she had been having difficulty taking her cysteamine on a consistent basis for the past few months due to multiple hospitalizations for other medical reasons. After our consultation, her medical condition remained stable for the next few months without any prolonged hospitalizations and she was able to continue her out-patient cysteamine treatment with minimal interruptions. On our next follow-up visit we observed an improvement in her thrombocytopenia with platelets increasing from 38×10^3^/μl to 60×10^3^/μl over the course of the next few months. Unfortunately, during the course of the next 2 years she had repeated hospital admissions for other medical problems and had much difficulty again maintaining consistent cysteamine treatment. Her thrombocytopenia worsened again to about 30×10^3^/μl. A repeat bone marrow biopsy was done (about 3 years after the initial bone marrow biopsy) and showed increased deposits of cystine crystals making up approximately 40% of her bone marrow components.

## Discussion

Here we present an adult patient with cystinosis who developed bone marrow suppression secondary to cystine crystal deposition. In cystinosis, the initial presenting symptoms occur approximately at 6 to 12 months of age with renal Fanconi’s syndrome, which is secondary to accumulation of cystine crystals in the kidneys causing proximal renal tubule damage [1, 10]. These patients develop dysfunction in the proximal renal tubules and are unable to reabsorb solutes [1, 10]. This leads to excessive urinary loss of protein, glucose, amino acids, phosphate, calcium, magnesium, sodium, potassium, bicarbonate, carnitine, and water [1, 10]. As a result these patients will clinically present with polyuria, polydipsia, electrolyte imbalance, dehydration, rickets, and growth failure if untreated as an infant [1, 10]. Prior to the 1970s cystinosis used to be a universally fatal pediatric disease due to renal failure secondary to renal Fanconi’s syndrome [1]. However, with the development of renal transplantation and cysteamine treatment this disease has been transformed into a chronic adult disease with multiorgan involvement [11]. Renal transplantation was first successfully performed in these patients in 1977 and has become widespread since then [11]. Around this time, cysteamine (beta-mercaptoethylamine) was found to deplete cultured cystinotic skin fibroblasts of cystine and has now become the standard of care [1]. Cysteamine works by reacting with cystine trapped in the lysosome and forms a mixed disulfide bond which allows it to exit the lysosome via the lysine (cationic) transport system [12]. However, cysteamine has many adverse effects including gastrointestinal toxicities such as nausea, vomiting, diarrhea, and a noxious rotten-egg odor and a taste that makes compliance very difficult [13]. In fact this drug’s gastrointestinal toxicities have been used as a model system to induce duodenal ulcers in rats for researchers [14]. Patients that manage to tolerate chronic cysteamine treatment have been observed to have a mean life expectancy of 28.5 years [1]. During their adult years, these patients have been found to develop extrarenal systemic involvement with end-organ damage in the eyes, muscles, central nervous system, and endocrine system [1]. Only in rare cases has it been observed to involve the bone marrow and cause clinical symptoms [4–9].

At the time of this publication a literature review shows only a handful of case reports describing pancytopenia in patients with cystinosis [4–9]. Pancytopenia is a medical condition with a large differential diagnosis that encompasses many different organ systems. It can be caused by various etiologies such as vitamin deficiencies secondary to gastrointestinal malabsorption, rheumatologic, infectious, and hematologic diseases. The cause is often multifactorial and requires a systematic work up in order to determine the root cause. In our patient she had a complicated medical history with many medical comorbidities encompassing multiple organ systems. This required us to perform a comprehensive work up to rule out common causes of secondary pancytopenia (infectious, vitamin deficiencies secondary to gastrointestinal malabsorption, rheumatologic, hematologic) as discussed in the Case presentation section.

As for possible hematologic diseases which could cause pancytopenia, the differential diagnosis in this patient included congenital and acquired disorders. For congenital disorders the differential diagnosis included Fanconi anemia, dyskeratosis congenita, Shwachman–Diamond syndrome, and congenital amegakaryocytic thrombocytopenia [15]. These diseases normally present in the early years of childhood before 10 years of age [15]. Fanconi anemia is an autosomal recessive or X-linked disorder with median age of presentation from 6- to 9-years old [15]. In addition, patients with pancytopenia often have clinical characteristics of short stature, hypopigmented and café-au-lait spots, abnormality of the thumbs, microcephaly or hydrocephaly, hypogonadism, and developmental delay [15]. Dyskeratosis congenita is characterized by bone marrow failure, cancer predisposition, and clinical characteristics demonstrating ectodermal dysplasia [15]. Classically, these patients were recognized with a triad of: 1) mottled skin hyperpigmentation of the face, neck, shoulders, and trunk, 2) nail dystrophy present bilaterally in the hands and feet, and 3) mucosal leukoplakia [15]. Approximately 70% of these patients will have one of the above physical examination findings [15]. Shwachman–Diamond syndrome usually presents in infancy and is characterized by exocrine pancreatic dysfunction, bone marrow failure, and skeletal abnormalities such as osteopenia leading to pathologic fractures [15]. Congenital amegakaryocytic thrombocytopenia is characterized by isolated thrombocytopenia in infancy with absent or a significantly reduced number of megakaryocytes out of proportion to the overall cellularity seen on bone marrow biopsy [15]. These patients are also noted to have none of the physical characteristics or birth defects associated with other inherited bone marrow failure syndromes [15]. As mentioned in the Case presentation section, our patient presented with pancytopenia at the age of 26 and she did not have any of the physical examination findings characteristic of the various congenital bone marrow failure syndromes. Furthermore, her bone marrow biopsy showed megakaryocytes which were present and not significantly reduced when considering the overall hypocellularity of the examined specimen. As a result we were able to conclude that her pancytopenia was very unlikely to be caused by one of the above congenital hematological disorders.

For acquired hematological disorders which could cause pancytopenia the differential diagnosis includes aplastic anemia, hematologic malignancies, and myelodysplastic syndrome. Aplastic anemia is characterized by diminished or absent hematopoietic precursors in the bone marrow, most often due to injury to the hematopoietic stem cells from primary or secondary causes. It is commonly a diagnosis of exclusion with bone marrow biopsy showing: 1) profound hypocellularity with decrease in all cellular lineages and an empty marrow composed mostly of fat cells and stroma, 2) no presence of infiltration from malignant cells or fibrosis, and 3) morphologically normal residual hematopoietic cells and hematopoiesis which is not megaloblastic. As mentioned above, our patient’s bone marrow biopsy did not show any immature or abnormal cells seen in hematological malignancies. Although the marrow was profoundly hypocellular in this patient, it did not appear to have a significantly increased deposition of fat cells or stroma as seen in aplastic anemia. In fact it appeared that a significant amount of marrow space (30% of the examined specimen) was replaced with the rhomboid and rectangular shaped, birefringent cystine crystals (Fig. 1). Therefore, these findings made it most likely that our patient’s pancytopenia was primarily due to myelosuppression secondary to bone marrow replacement from deposition of cystine crystals. However, we acknowledge that since aplastic anemia is a diagnosis of exclusion with no clinically available specific test to rule it in or out we cannot exclude the possibility that the patient may also have an additional underlying superimposed aplastic anemia.

## Conclusions

In conclusion, pancytopenia secondary to myelosuppression from cystine crystal deposition in the bone marrow is a very rare complication. This case illustrates the importance of keeping a broad differential diagnosis and systematically ruling out common causes of pancytopenia. It also demonstrates the importance of bone marrow biopsies in the evaluation of unexplained pancytopenia.

## Consent

Written informed consent was obtained from the patient for publication of this case report and any accompanying images. A copy of the written consent is available for review by the Editor-in-Chief of this journal.

## Abbreviations

CKD: chronic kidney disease
DVT: deep vein thrombosis
ESRD: end-stage renal disease
WBC: white blood cell
